# Active-touch texture/material matching and caregiver-reported sensory reactivity in adolescents with autism spectrum disorder: a pilot study

**DOI:** 10.3389/fpsyt.2026.1826858

**Published:** 2026-06-01

**Authors:** Yukie Chounan, Kyoko Suzuki

**Affiliations:** 1 Mie Prefectural College of Nursing, Tsu, Japan; 2 Department of Behavioral Neurology and Cognitive Neuroscience, Tohoku University Graduate School of Medicine, Sendai, Japan; 3 Support Center for Acquired Cognitive and Behavioral Disorders, Tohoku Medical and Pharmaceutical University Hospital, Sendai, Japan

**Keywords:** active touch, adolescence, autism spectrum disorder, sensory assessment, sensory reactivity, tactile perception

## Abstract

This pilot study examined whether caregiver-reported sensory characteristics were associated with basic somatosensory functions and tactile texture/material matching performance during active touch. Thirteen adolescents with clinically diagnosed ASD and 13 age-matched typically developing (TD) controls participated in the study. Somatosensory function was evaluated using finger kinesthesia, two-point discrimination, and graphesthesia as indices of somatosensory processing. Texture/material matching performance was assessed using a tactile–visual texture/material matching task. Caregiver-reported sensory features were obtained using the Japanese version of the Sensory Profile (SP-J). As expected, the ASD group differed from the TD group in SP-J sensory behavior ratings. However, active-touch texture/material matching performance was not significantly different between groups. Moreover, texture/material matching performance was not consistently associated with caregiver-reported sensory characteristics, including tactile reactivity and sensory processing quadrants. These preliminary findings suggest that what caregivers describe as sensory hyper- or hypo-reactivity in everyday situations may not be fully explained by objectively measured texture/material matching performance during active touch. One possible explanation is that active touch may allow individuals to regulate contact with textures, whereas daily experiences also include less controllable passive tactile input. Objective assessment of tactile processing may help complement caregiver-reported sensory measures in characterizing tactile function in adolescents with ASD.

## 1 Introduction

Atypical sensory reactivity (i.e., hyper- and/or hypo-responsiveness to sensory stimuli) is a well-established feature of autism spectrum disorder (ASD) (1–3). Sensory hyper- or hypo-reactivity is now included as a core diagnostic feature in the DSM-5, underscoring its clinical significance (4). Parent reports of everyday sensory symptoms indicate that sensory atypicalities are observed in approximately 69% of children with ASD (5). Recent reviews have further highlighted the importance of tactile abnormalities within the sensory features of ASD. For example, atypical tactile sensitivity in ASD may be reflected in heightened discomfort, negative emotional responses, or avoidance during tactile encounters with specific textures or materials (6, 7). These sensory features may not diminish with age (8), and in some individuals they may even worsen (9). Thus, sensory features in ASD are considered persistent across the lifespan and can substantially impact everyday functioning. In the present study, sensory sensitivity and tactile reactivity refer to subjective and behavioral responses to tactile stimuli in everyday contexts, such as discomfort or distress.

Among sensory domains, tactile processing is relevant in both home and school contexts. Compared with home environments, more pronounced tactile responses have been reported in school settings (10). Tactile reactivity may contribute to reduced task engagement and learning-related concentration (11), influence interpersonal interactions (12), and affect broader social functioning (13). Given that classroom environments require sustained attention and frequent physical contact with objects and peers, atypical tactile processing may disproportionately affect adolescents in educational settings.

Behavioral manifestations of tactile atypicalities in ASD may be associated with differences in neural processing of touch. Functional magnetic resonance imaging (MRI) studies have shown greater neural responses to mildly aversive tactile and auditory stimuli in individuals with ASD, as well as reduced neural habituation to repeated stimulation in those with tactile hypersensitivity (14). In addition, somatosensory evoked potential studies report no group differences in early components (P50, N80, P100), but shorter latencies in the later N140 component, suggesting differences in later stages of tactile processing (15). These findings collectively suggest that atypical tactile experiences in ASD may arise not only from peripheral sensitivity but also from altered central sensory integration and higher-order processing. However, objective psychophysical findings regarding lower-level tactile processing in ASD remain inconsistent.

Some studies have reported elevated detection or temporal discrimination thresholds, whereas others have found no clear group differences in tactile resolution or detection performance (16–21). Previous studies have suggested that responses to vibrotactile tasks may not necessarily correspond to questionnaire-based sensory features reported by caregivers or individuals themselves. Together, these findings suggest that subjective tactile reactivity, physiological responses, and objective perceptual performance may capture related but not identical aspects of tactile functioning. Accordingly, tactile behaviors suggestive of sensory hyper- or hypo-reactivity in ASD may not be explained solely by primary somatosensory processing and may also involve higher-order tactile cognition.

Beyond low-level tactile processing, higher-order tactile functions such as texture and material perception may also be related to everyday sensory reactivity in ASD. While some studies report no group differences in roughness discrimination (22), others suggest that adults with ASD may perceive textures as rougher than typically developing (TD) adults (23). Importantly, the relationship between tactile texture/material perception and everyday sensory reactivity remains largely unexplored. Furthermore, while previous studies on tactile texture perception have primarily focused on children or adults (22–25), higher-order tactile texture recognition in adolescents remains insufficiently understood. Previous studies also suggest that higher-order tactile processing in ASD may vary across developmental stages. For example, adults with ASD showed superior haptic-to-visual shape matching during active touch (26), whereas children with ASD showed poorer tactile processing than typically developing peers (27). Moreover, differences in perceptual and neural responses to tactile texture stimulation in ASD have been reported (25), and studies have examined visuo-tactile integration in children and adolescents with ASD (28). However, studies specifically investigating active tactile perception in adolescence remain limited. Given this gap, the present study focused on adolescents.

Tactile texture processing can be examined under both active and passive touch conditions. Active touch refers to situations in which individuals voluntarily move their hands to explore an object or surface, whereas passive touch refers to situations in which tactile stimuli are applied to a stationary hand, finger, or body part. Previous studies of tactile texture processing in ASD have examined roughness perception, affective evaluations, or preferences under active and passive touch conditions. For example, one study of active roughness perception reported that adults with ASD rated textured surfaces as rougher and showed greater trial-to-trial variability than controls (23). Another active-touch study asked 10 adults with ASD to touch seven types of fabric, including satin, denim, hessian, cotton, polyester, wool, and spandex, and primarily examined their subjective tactile experiences, fabric preferences, and the perceived impact of fabrics on daily life (29). Nevertheless, this qualitative study did not include an objective task assessing whether participants could accurately recognize, discriminate, or match the textures or materials. In a passive-touch study, eight adults with high-functioning ASD and eight typically developing controls were touched with three types of materials, including hessian, plastic mesh, and a soft brush, and were asked to rate pleasantness (24). Although these studies have provided important information regarding tactile sensitivity, roughness perception, tactile preference, and affective responses to tactile stimuli in ASD, direct assessment of texture/material recognition itself remains limited. This distinction is important because roughness ratings and affective evaluations may reflect subjective perceptual or emotional responses to tactile stimuli, whereas texture/material matching requires perceptual-cognitive recognition of tactile material properties during active exploratory touch. To our knowledge, no previous study has directly examined whether performance in active-touch texture/material recognition is associated with caregiver-reported sensory reactivity in adolescents with ASD. Therefore, texture/material recognition during active touch in adolescents with ASD remains insufficiently understood.

To examine whether higher-order tactile processing is related to caregiver-reported sensory characteristics in ASD, the present study assessed active-touch texture/material matching together with basic somatosensory functions, including finger kinesthesia (judgment of the direction of passive finger movement), graphesthesia (ability to recognize numbers or symbols traced on the skin), and two-point discrimination (ability to distinguish two simultaneous tactile stimuli as separate points). These basic somatosensory measures were included to help determine whether texture/material matching performance could be explained by lower-level somatosensory abilities or reflected aspects of higher-order tactile recognition. Adolescents with ASD and age-matched TD controls completed an active-touch texture/material matching task, and associations with caregiver-reported sensory features measured using the Japanese version of the Sensory Profile (SP-J; 30) were examined. The primary outcome was performance on the texture/material matching task; secondary outcomes included finger kinesthesia, graphesthesia, two-point discrimination, and SP-J scores. We hypothesized that adolescents with ASD would differ from TD controls in active-touch texture/material matching accuracy. We further conducted exploratory analyses to examine associations between texture/material matching performance and caregiver-reported sensory characteristics, including tactile reactivity and sensory processing quadrants.

## 2 Methods

### 2.1 Participants

Participants with ASD were recruited through a prefectural branch of a parents’ association for families of children with developmental disabilities, and through outpatient clinics serving children with developmental disabilities. Inclusion criteria for the ASD group were (1): a clinical diagnosis of ASD based on DSM-IV or DSM-5 criteria (confirmed via diagnostic documentation; International Statistical Classification of Diseases and Related Health Problems, 10th Revision (ICD-10) codes were checked as needed) (2); age 12–18 years (3); Full Scale IQ ≥ 70 on the Wechsler Intelligence Scale for Children (WISC-III/IV), confirmed using the most recent result (4); vision and hearing adequate to complete the assessments; and (5) written informed consent obtained from both the adolescent and a parent/guardian. The TD group was recruited via public postings in a governmental information/exchange center and via snowball recruitment through acquaintances. Eligibility for TD participants was enrollment in regular classrooms without special support services, age 12–18 years, and vision and hearing adequate to complete the assessments. Written informed consent was obtained from both the adolescent and a parent/guardian in both groups. To screen for elevated autistic traits, parents completed the Japanese version of the Social Responsiveness Scale, Second Edition (SRS-2; 31). Formal IQ testing was not conducted in the TD group. In the ASD group, comorbid ADHD was recorded based on a prior clinical diagnosis. By contrast, the Conners 3-P was administered to assess ADHD-related traits dimensionally and was not used to determine ADHD comorbidity.

Among the 17 ASD applicants, four were excluded (two with Full Scale IQ < 70; one unreachable; one outside the target age range). The final ASD sample comprised 13 adolescents (mean age 15.56 ± 1.96 years), including 6 males and 7 females; 6 participants had a comorbid clinical diagnosis of ADHD. Among the 16 TD applicants, three were excluded because of elevated autistic traits assessed via the parent-rated SRS-2: total T-score ≥ 60 (a standardized score with a mean of 50 and a standard deviation of 10) was the recommended cutoff (31). The final TD sample comprised 13 adolescents (mean age 15.01 ± 1.87 years), including 3 males and 10 females. The study protocol received approval from the institutional ethics committee of the participating institution and was conducted in accordance with the Declaration of Helsinki.

### 2.2 Procedure

All testing was conducted in a quiet, private research room. Participants were seated and underwent three somatosensory tasks and the active-touch texture/material matching task. Participants were blindfolded during the somatosensory tasks. During the tactile texture/material matching task, participants selected their responses from a visual reference set of the same materials.

#### 2.2.1 Somatosensory tasks

All somatosensory tasks were administered by the same examiner using a standardized procedure across participants, based on standard neurological examination methods (32). Somatosensory functions were assessed using: (a) finger kinesthesia (judgment of the direction of passive finger movement); (b) graphesthesia (ability to recognize numbers or symbols traced on the skin); and (c) two-point discrimination (ability to distinguish two simultaneous tactile stimuli as separate points). Finger kinesthesia was assessed using a standard neurological examination procedure for deep sensation. Participants wore an eye mask and were asked to hold one hand horizontally with the palm facing downward. The examiner sat facing the participant and assessed the metacarpophalangeal joint of the index finger. To minimize tactile pressure cues, the examiner lightly held the sides of the finger and passively moved the joint slightly upward or downward. Participants were then asked to verbally report the perceived direction of movement (“up” or “down”). This task assessed the ability to judge the direction of passive finger movement as a clinical measure of proprioceptive function, rather than separately assessing position sense and movement sense, which are considered distinct aspects of proprioception (33). Five trials were administered for each hand, for a total of 10 trials, and the number of correct responses was recorded. One point was assigned for each correct response for each side; the total score was the sum of right and left performances (R + L; maximum 10 points). Graphesthesia was assessed to evaluate higher-order somatosensory function under conditions in which superficial sensation was preserved. Participants wore an eye mask while the examiner traced numbers on the palm, and participants were asked to identify the perceived number. Participants were not informed in advance that the possible stimuli were limited to the numbers “3,” “6,” and “9”; they were simply instructed to report the number traced on their palm. The examiner sat beside the participant and used a tablet stylus (digitizer stylus pen for Sony Vaio Duo 11) to trace the numbers on the participant’s palm. The stimuli consisted of the numbers “3,” “6,” and “9.” The size of each traced number was adjusted to each participant’s hand size so that the number extended across the central area of the palm. Participants responded verbally with the number they perceived. Five trials were administered for each hand, for a total of 10 trials. One point was assigned for each correct response, and the total score was calculated as the sum of right- and left-hand performance (R + L; maximum = 10 points). Graphesthesia testing was performed according to a standard neurological examination procedure for combined sensory function (32).

For two-point discrimination, participants wore an eye mask while the examiner used a Disk-Criminator (JAMAR, Discriminator, 52618, Patterson Medical Holdings Inc.) to apply one-point or two-point stimuli to the pulp of the index finger. Participants verbally reported whether they perceived “one point” or “two points.” Stimulus distances ranged from 1 to 8 mm in 1-mm increments and were presented in random order to evaluate performance across and beyond the expected fingertip discrimination threshold range. Two sets were administered for each hand, and the minimum distance (mm) at which two points were correctly identified as separate was used as the threshold for analysis. The test procedure was based on a standard neurological examination method for combined sensory function (32).

#### 2.2.2 Active-touch texture/material matching task

Stimuli consisted of eight 10 × 10 cm material squares (elastomer rubber, sandpaper, nonwoven sponge, hemp, artificial grass, velvet, plasticine, and slime). Selection was informed by prior tactile studies that used sandpaper and coarse fabric materials such as hessian (22, 24) and by practical relevance to everyday group activities. Materials were newly prepared for each participant to ensure consistency of tactile properties.

The task was administered behind a partition screen to prevent visual access to the stimulus.

Participants explored each material bimanually and verbally selected the matching item from eight visual references. To minimize participant burden and fatigue, particularly given the inclusion of multiple sensory assessments within a single session, each material was presented once, resulting in a total of eight trials. Participants were informed of this trial structure in advance. This decision was made to ensure feasibility and sustained engagement in adolescents with ASD, although the limited number of trials may have reduced the sensitivity of the task to detect subtle performance differences.

##### 2.2.2.1 Scoring

If the explored material and the selected material matched, 2 points were awarded. If the response was within a predefined “similar category,” 1 point was awarded; all other errors received 0 points. Similar categories were defined *a priori* based on shared tactile properties, particularly surface roughness and friction-related tactile qualities (e.g., rough/smooth, moist/dry, and sticky/slippery dimensions) (34). The predefined categories were as follows:

- “Prickly/rough–like” category: sandpaper, hemp, nonwoven sponge, artificial grass
- “Sticky/moist-like” category: slime, plasticine

This categorization was determined before data analysis. This scoring approach was adopted to capture not only exact material matching but also partial recognition of shared tactile qualities. Materials within the predefined similar categories were considered partially correct because they shared salient tactile properties, particularly roughness or friction-related tactile qualities. Thus, the scoring scheme allowed us to distinguish complete identification from partial texture/material recognition.

The summed score across eight trials was used as the active-touch texture/material matching score.

#### 2.2.3 Parent-report questionnaires

Parents completed questionnaires mailed prior to the laboratory visit and returned them on the test day. Measures included: Japanese version of the Sensory Profile (SP-J), the Japanese version of SRS-2, and the Japanese version of Conners 3rd Edition–Parent (Conners 3-P). Scoring followed the respective manuals (30, 31, 35). Clinical interpretation of scores followed the criteria indicated in the study’s **Table 1** note. Data collection occurred between May 2021 and December 2023. Participants and parents received a 3,000-yen voucher as compensation.

**Table 1:** Sensory Profile (SP-J) results.

	ASD (n=13)	TD (n=13)	U	*z*	*r*	*p*
	mean (SD)	mean (SD)				
Quadrants						
Low registration	34.23 (12.59)	16.23 (2.24)	0	-4.37	.86	<.001
Sensation seeking	46.69 (11.25)	27.92 (3.15)	4.5	-4.15	.81	<.001
Sensory sensitivity	39.46 (14.73)	23.46 (4.07)	10.5	-3.80	.75	<.001
Sensory avoiding	73.08 (22.21)	39.85 (8.19)	11.5	-3.75	.74	<.001
Sensory processing						
Touch processing	35.62 (14.12)	21.08 (4.75)	20.0	-3.37	.66	.001

Values are means (SD).

p values were obtained using the Mann–Whitney U test.

SP-J quadrant scores were interpreted according to the manual with reference to the normative range; deviation from the normative range may occur in either direction depending on the quadrant.

### 2.3 Statistical analysis

Primary, secondary, and exploratory outcomes were prespecified. The primary analysis compared groups on their total active-touch texture/material matching scores. Secondary analyses compared groups on somatosensory functions and SP-J Touch Processing and quadrant scores. Exploratory analyses characterized low-performing ASD cases (≤ TD mean − 1.5 SD) and their error patterns.

After compiling measures from the somatosensory tasks, the tactile texture/material matching task, and parent-report measures (SP-J quadrants and Touch domain, SRS-2, and Conners 3-P), group comparisons were conducted using the Mann–Whitney U test due to the small sample size. Group differences in categorical variables (sex) were examined using Fisher’s exact test. Hodges–Lehmann median differences with 95% confidence intervals were estimated for group comparisons.

Associations between texture/material matching performance and SP-J Touch Processing and quadrant scores were examined separately within the ASD and TD groups using Spearman’s rank correlation coefficients. For correlation analyses, 95% confidence intervals were estimated using 1,000 bootstrap resamples.

To control for potential inflation of Type I error due to multiple comparisons, false discovery rate (FDR) correction using the Benjamini–Hochberg procedure was applied to the families of correlation analyses within each group. The overall pattern of results remained unchanged after correction.

Effect sizes (r for Mann–Whitney U tests and Spearman’s ρ for correlations) were reported to facilitate interpretation. Given the modest sample size, emphasis was placed on effect sizes and confidence intervals rather than sole reliance on p-values. Analyses were performed using SPSS version 29.0 with a two-tailed alpha of 0.05.

## 3 Results

### 3.1 Participant characteristics, social responsiveness, and ADHD-related traits

No significant group differences in age were observed between ASD (15.56 ± 1.96 years) and TD (15.01 ± 1.87 years; p = .511; see **Table 2**). Sex distribution did not differ significantly between groups (male: ASD 6 vs. TD 3; p = .411). In the ASD group, six participants had a comorbid clinical diagnosis of ADHD.

**Table 2:** Participant demographics and questionnaire scores.

	ASD n=13	TD n=13	U	z	*r*	*p*
Age (years)	15.56 (1.96)	15.01 (1.87)	71.5	-0.67	.13	.511
Sex (male/female)	6/7	3/10	―	―	―	.411
ADHD comorbidity	6	0	―	―	―	―
SRS-2 (Japanese version)						
Social Communication and Interaction: SCI	73.31 (14.72)	45.62 (6.5)	162.00	3.98	.78	<.001
Restricted Interests and Repetitive Behavior: RRB	81.23 (13.50)	47.77 (8.36)	163.50	4.11	.81	<.001
Total T-score	77.23 (14.21)	45.85 (6.36)	162.00	3.99	.78	<.001
Social awareness	64.08 (12.15)	46.77 (10.05)	146.00	3.16	.62	.002
Social cognition	72.69 (15.34)	43.38 (6.38)	159.50	3.85	.76	<.001
Social communication	73.15 (14.56)	45.62 (8.67)	158.00	3.78	.74	<.001
Social motivation	68.38 (14.96)	48.92 (7.96)	146.00	3.17	.62	.002
Conners 3-P (Japanese version)						
Inattention	70.00 (14.62)	46.23 (7.79)	156.00	3.67	.72	<.001
Hyperactivity/Impulsivity	66.31 (17.88)	47.85 (8.17)	139.50	2.82	.55	.003
ADHD inattention symptoms	72.46 (13.31)	46.38 (7.76)	160.00	3.88	.76	<.001
ADHD hyperactivity/impulsivity symptoms	66.00 (17.42)	47.08 (6.28)	141.50	2.93	.57	.002

Values are means (SD).

p values for sex were obtained using Fisher’s exact test.

p values were obtained using the Mann–Whitney U test.

Group comparison was not performed because ADHD diagnosis was specific to the ASD group.

“―” indicates not tested.

ASD, autism spectrum disorder; TD, typically developing; ADHD, attention-deficit/hyperactivity disorder; SRS-2, Social Responsiveness Scale, Second Edition; Conners 3-P, Conners Third Edition–Parent Rating Scale.

On the Japanese SRS-2, the ASD group had significantly higher total T-scores and higher scores across all subscales compared with the TD group (all p <.001). Similarly, on the Japanese Conners 3-P, the ASD group demonstrated significantly higher scores for Inattention, Hyperactivity/Impulsivity, and all ADHD symptom-related indices (all p <.01).

### 3.2 Active-touch texture/material matching performance and somatosensory functions

On the active-touch texture/material matching task, no significant differences were observed between ASD and TD groups (p = .287; see **Table 3**), with mean scores of 14.54 ± 1.66 in the ASD group and 15.15 ± 1.63 in the TD group. The Hodges–Lehmann estimated median difference between groups was 0 (95% CI [0, 2]). Both groups achieved the maximum scores on the finger kinesthesia (R + L = 10), resulting in no variance and precluding group comparisons for that measure. Graphesthesia scores did not differ between groups (ASD: 9.38 ± 1.45; TD: 9.63 ± 0.87; p = .960). Two-point discrimination thresholds also did not show a statistically significant group difference (ASD: 2.54 ± 0.52 mm; TD: 3.00 ± 0.71 mm; p = .091).

**Table 3:** Active-touch texture/material-matching and somatosensory function.

	ASD (n=13)	TD (n=13)	HL diff [95% CI]	U	*z*	*r*	*p*
Somatosensory functions							
Index-finger kinesthesia (R+L, Max 10)	10	10	N/A	N/A	N/A	N/A	N/A
Graphesthesia (R+L, Max 10)	9.38 (1.45)	9.63 (0.87)	0 [0, 0]	83.00	-.10	.02	.960
Two-point discrimination (min threshold, mm)	2.54 (0.52)	3.00 (0.71)	0 [0, 1]	51.50	-1.76	.35	.091
Active-touch texture/material matching (Max 16)	14.54 (1.66)	15.15 (1.63)	0 [0, 2]	106.00	1.27	.25	.287

p values were obtained using the Mann–Whitney U test.

N/A: not applicable because all values were identical across groups.

HL diff indicates the Hodges–Lehmann estimated median difference between groups with 95% confidence intervals.

### 3.3 The Japanese version of the Sensory Profile

On the SP-J, the ASD group demonstrated significantly higher scores than the TD group on all four quadrants (Low Registration, Sensation Seeking, Sensory Sensitivity, and Sensation Avoiding; all p <.001; see **Table 1**). The Touch sensory processing domain score was also significantly higher in the ASD group (p = .001), reflecting higher parent-reported atypicalities in tactile-related sensory responses.

### 3.4 Associations between tactile matching performance and SP-J

Within-group analyses showed no significant correlations between tactile matching performance and SP-J Touch Processing and quadrant scores in either group (all |ρ| ≤.30, all p ≥.31), and none remained significant after FDR correction (see **Supplementary Table 1**).

### 3.5 Exploratory description of low performance and error patterns

Although no group-level difference was observed, exploratory analyses identified three ASD participants with a low tactile matching performance (defined *a priori* as ≤ TD mean − 1.5 SD). Across these participants, two-point discrimination thresholds were within normal range (minimum threshold = 3 mm). In addition, two of the low-performing cases showed no apparent abnormalities on finger kinesthesia or graphesthesia testing, whereas one case showed reduced graphesthesia performance. These findings suggest that low performance on the active-touch texture/material matching task was not clearly accompanied by abnormalities in other somatosensory tasks. On the texture/material matching task, two of these participants showed reciprocal confusion between elastomer rubber and plasticine, and the third showed the same confusion plus misidentification of velvet as plasticine.

The SP-J profiles varied across these individuals. All three low-performing ASD cases showed elevated low-registration scores. Two cases also showed high scores in multiple domains, including sensation seeking, sensation avoiding, and tactile-related scores, whereas the remaining case was characterized by particularly elevated sensory sensitivity. Thus, the SP-J patterns were not uniform across the low-performing cases.

## 4 Discussion

This study examined whether active-touch texture/material matching performance, as a task-based measure of tactile perceptual-cognitive processing, is associated with caregiver-reported sensory features in adolescents with ASD. The principal findings were: (1) no significant ASD–TD group differences in active-touch texture/material matching performance or in somatosensory function (graphesthesia and two-point discrimination); and (2) only nonsignificant correlations were observed between tactile matching performance and SP-J indices (quadrants and Touch domain) in both groups. In contrast, the ASD group showed markedly higher parent-reported sensory atypicalities on SP-J across quadrants and tactile processing, which is consistent with prior literature indicating prominent sensory features in ASD (1–3, 5, 7).

The ASD group did not show a significant decrease in active-touch texture/material matching. While previous studies examined simple roughness-related or affective tactile discrimination tasks and reported no clear difference between ASD and TD groups (22, 24, 25), the present study found no significant group difference in performance on the higher-order texture/material matching task. Previous work has reported intact or even superior haptic-to-visual shape matching performance in individuals with ASD (26), suggesting that cross-modal transformation from touch to vision may be preserved or enhanced in certain perceptual domains. However, such findings in shape-based tasks do not necessarily generalize to texture or material recognition. Shape recognition primarily depends on the integration of global spatial configuration, whereas texture and material recognition rely more heavily on fine-grained surface properties and dynamic material cues (e.g., roughness, compliance, friction). These differences may involve distinct patterns of sensory integration, which could contribute to domain-specific variations in haptic processing in ASD.

The present results therefore suggest that haptic perceptual processing in ASD may vary depending on the specific perceptual dimension being assessed. Although ADHD-related traits were elevated in the ASD group, the absence of group differences in tactile matching performance does not provide clear evidence that elevated ADHD-related traits impaired task performance at the group level. However, because ADHD-related traits and comorbid ADHD were not sufficiently disentangled in the present sample, their possible influence should be interpreted cautiously. In particular, prior work has suggested that tactile processing profiles may differ depending on the presence of ADHD (36). Therefore, some of the tactile characteristics observed in our ASD sample may partly reflect the influence of ADHD comorbidity. However, given the limited sample size, particularly the small number of ASD participants with comorbid ADHD, the present study could not determine whether the observed pattern primarily reflected ASD-related features, ADHD-related traits/comorbidity, or their overlap.

Under an active-touch condition—where participants can modulate contact pressure, exploration speed, and strategy—it may be difficult to detect a uniform reduction of tactile texture/material identification accuracy as a group trait in ASD. Exploratory descriptions suggested variability within the ASD group. The presence of low-performing participants (≤ TD mean − 1.5 SD) and a shared error pattern—reciprocal confusion between elastomer rubber and plasticine—may suggest that some individuals may have difficulty with tactile material identification when discriminative cues are subtle. In these three cases, two-point discrimination thresholds were preserved, suggesting that low performance was not readily explained by lower-level somatosensory function alone. Differences in higher-order processes (e.g., feature extraction/integration) and/or exploration strategies during active touch may contribute to performance variability. Moreover, because haptic object identification shows substantial developmental improvement by late childhood in typically developing individuals (37), developmental immaturity alone is unlikely to fully account for the observed low performance.

Taken together, these observations suggest that material identification for adolescents with ASD may become more difficult under haptic conditions in which discriminative cues such as surface roughness and friction-related tactile qualities are relatively subtle, such that differences in exploratory strategies are more likely to be reflected in task performance. Furthermore, because responses were selected from a visual reference set after tactile exploration, we cannot rule out the possibility that individual differences in visual material/texture processing influenced performance. Future studies should use stimuli that systematically manipulate discriminative cues (e.g., surface roughness, elasticity, and friction-related tactile qualities) across graded levels and include multiple trials, while also considering individual differences in visual material/texture processing, to clarify the conditions under which misidentification is most likely to occur and identify the underlying factors. Such refinements may also help reduce possible ceiling effects and improve sensitivity to subtle group differences.

The absence of statistically significant associations between tactile matching performance and SP-J Touch Processing score or other SP-J indices may not be surprising, given that parent-reported sensory reactivity in daily life (e.g., sensitivity and avoidance) and performance in a controlled, active-touch identification task are likely to reflect partially distinct aspects of tactile functioning. This pattern may be consistent with a partial dissociation between affective-reactive responses to everyday tactile experiences and perceptual-cognitive identification processes under self-regulated active exploration in ASD.

In everyday contexts, tactile difficulties often arise during passive or unpredictable contact (e.g., incidental touch by others and unexpected textures), which may provoke emotional or defensive responses (12). One possible explanation, not directly tested in the present study, is that active touch may allow participants to regulate contact style, which could reduce the impact of tactile discomfort and support compensatory exploration strategies. Thus, questionnaire-based indices of sensory sensitivity/avoidance and task-based measures of tactile identification may not be directly aligned, as they capture partially distinct components: affective reactivity and contextual intolerance versus perceptual/cognitive identification under self-controlled exploration. In addition, subjective tactile experiences such as discomfort or irritation, as well as physiological responses, may be more directly relevant to everyday sensory difficulties than task-based identification performance alone. This may be particularly important during adolescence and older childhood, when first-person reports can provide valuable complementary information about sensory experiences that may not be fully captured by caregiver reports or behavioral tasks. At the same time, task-based perceptual assessment may still provide complementary information regarding the underlying tactile processing that may be relevant to such everyday sensory experiences.

The observed divergence between laboratory-based tactile performance and parent-reported tactile reactivity highlights the need for individualized characterization of tactile functioning that considers both subjective reports and objective perceptual measures.

## 5 Limitations and future directions

Several limitations should be noted. First, the sample size was small; therefore, the findings should be interpreted as preliminary and require replication in larger cohorts. The small sample size raised the possibility of insufficient statistical power to detect subtle group differences or correlations. Although false discovery rate correction was applied, the modest sample size may have increased the risk of Type II error. Given the well-documented heterogeneity of sensory profiles in ASD, future studies with larger samples should consider defining subgroups based on tactile behavioral profiles to examine whether distinct tactile-cognitive patterns correspond to these subgroups.

Second, the number of trials or responses was limited across the behavioral measures. In the texture/material matching task, each material was presented only once, which restricted reliability and limited the ability to average intra-individual variability. Although the basic somatosensory tasks were administered according to standard neurological examination procedures, they were not optimized as quantitative psychophysical measures and therefore may have had limited sensitivity to subtle individual or group differences. Because performance was high in both groups, the limited number of trials or responses and relatively constrained task difficulty may have contributed to ceiling effects, particularly in the texture/material matching and basic somatosensory tasks. Although increasing the number of trials may improve reliability and sensitivity, balancing methodological rigor with participant burden may be particularly important when administering repetitive tactile tasks to adolescents with ASD in clinical research settings. Future studies should therefore aim to optimize both the number of trials and the feasibility of task administration, while also evaluating the reliability of these measures. Although the somatosensory tasks were administered using standardized neurological examination procedures, formal inter-rater or test–retest reliability was not assessed in the present study. The finger kinesthesia used in this study was based on a clinical neurological examination in which participants judged the direction of passive finger movement; therefore, it did not separately assess position sense and movement sense. Because participants selected their responses from a visual reference set after tactile exploration, we could not rule out the influence of individual differences in visual material or texture processing on task performance.

Third, this study assessed tactile recognition only under an active-touch condition; passive-touch responses that may better reflect unpredictable real-world tactile encounters should also be examined. Another limitation is that subjective tactile experiences were not assessed directly from the participants themselves. Future studies should incorporate participant-reported sensory measures, such as self-report sensory profile questionnaires, particularly in adolescents, to assess subjective tactile experiences.

The potential influence of ADHD-related traits and comorbid ADHD should also be considered. Because only 6 of the 13 adolescents in the ASD group had a comorbid clinical diagnosis of ADHD, subgroup analyses comparing ASD participants with and without ADHD would have lacked sufficient statistical power. Thus, the present study could not determine whether the observed pattern of tactile task performance reflected ASD-related features, ADHD-related traits/comorbidity, or their overlap. Accordingly, the present findings should be interpreted cautiously with regard to the specific contribution of ADHD-related traits. Mechanistic conclusions regarding higher-order tactile processing should be considered preliminary. This issue is particularly relevant in light of prior work showing disorder-specific alterations of tactile sensitivity across ASD, ADHD, ASD + ADHD, and typically developing groups (36). Thus, some of the tactile characteristics observed in our ASD sample may partly reflect the influence of ADHD comorbidity.

## 6 Conclusions

In this preliminary study, adolescents with ASD did not show statistically significant differences in active-touch texture/material matching or somatosensory function despite elevated caregiver-reported tactile hyper- or hypo-reactivity in everyday contexts. These findings suggest that task-based measures of perceptual-cognitive tactile processing and caregiver-reported sensory reactivity may capture partially distinct aspects of tactile functioning. Task-based assessments may therefore provide complementary information to caregiver-reported and self-reported sensory measures when characterizing tactile function in adolescents with ASD.

## Data Availability

Individual-level data are not publicly available due to ethical and privacy restrictions, including the risk of re-identification in a small sample of minors. In addition, the informed consent obtained from participants and their guardians did not permit data sharing beyond the scope of the present study. Any inquiries will be considered only within these restrictions. Requests to access the datasets should be directed to yuchounan@gmail.com.
