# Supplementary material for: Active-touch texture/material matching and caregiver-reported sensory reactivity in adolescents with autism spectrum disorder: a pilot study
Source: Front Psychiatry. 2026 Jun 1;17:1826858. doi: 10.3389/fpsyt.2026.1826858 (PMC13265568; doi:10.3389/fpsyt.2026.1826858)
Supplement: Supplementary file 1 [file Table1.docx]

Supplementary Table S1. Spearman correlations between active-touch tactile texture/material matching and SP-J Touch processing and quadrant scores in the ASD and TD groups.

|  | ASD | | | TD | | |
| --- | --- | --- | --- | --- | --- | --- |
| SP-J Subscale | ρ | p | 95% CI | ρ | p | 95% CI |
| Low Registration | .043 | .890 | [−.51, .61] | -.200 | .512 | [−.73, .31] |
| Sensation Seeking | -.135 | .661 | [−.64, .47] | -.304 | .312 | [−.84, .33] |
| Sensory Sensitivity | .295 | .327 | [−.26, .77] | -.161 | .600 | [−.75, .53] |
| Sensory Avoidance | -.108 | .724 | [−.58, .47] | -.041 | .894 | [−.65, .57] |
| Touch Processing | -.003 | .992 | [−.51, .52] | -.289 | .338 | [−.86, .48] |

Spearman’s rank correlations are reported.

95% confidence intervals were estimated using 1,000 bootstrap resamples.

False discovery rate (FDR) correction was applied separately within each group; no correlations remained significant.
